# Clip-On IMU System for Assessing Age-Related Changes in Hand Functions

**DOI:** 10.3390/s20216313

**Published:** 2020-11-05

**Authors:** Seungjae Lee, Hyejeong Lee, Jongshill Lee, Hokyoung Ryu, In Young Kim, Jieun Kim

**Affiliations:** 1 Department of Biomedical Engineering, Hanyang University, Seoul 04763, Korea; seungjaelee@hanyang.ac.kr (S.L.); netlee@hanyang.ac.kr (J.L.); iykim@hanyang.ac.kr (I.Y.K.); 2 ImagineXlab, Hanyang University, Seoul 04763, Korea; lhj4625@hanyang.ac.kr (H.L.); hryu@hanyang.ac.kr (H.R.)

**Keywords:** hand and finger kinematics, inertial measurement units, targeted box and block test, real-time motion, hand function assessment

## Abstract

Hand functions affect the instrumental activities of daily living. While functional outcome measures, such as a targeted box and block test, have been widely used in clinical settings and provide a useful measure of overall performance, the advent of a wearable Inertial Measurement Unit(IMU)-based system enables the examination of the specific performance and kinematic parameters of hand movements. This study proposed a novel clip-on IMU system to facilitate the clinically fitted measurements of fine-motor finger and wrist joint movements. Clinical validation was conducted with the aim of characterising age-related changes in hand functions, namely grasping, transporting, and releasing blocks. Eighteen young (age 20–31) and sixteen healthy older adults (age 75–89) were evaluated during the box and block test. The results demonstrated that an older age was characterized by slower movements and higher variations and kinematic alterations in the hand functions, such as a larger range of motions at the fingers as well as kinematic trajectories. The proposed IMU system and subsequent validations highlight the value of the performance and kinematics parameters for a more comprehensive understanding of fine-motor finger and wrist movements that could shed light on further implementations in clinical and practical settings.

## 1. Introduction

Hand function plays a fundamental role in the activities of daily living. The ability to manipulate everyday objects requires complex hand movements, such as grasping, touching, and lifting [1]. Factors associated with ageing and chronic diseases (e.g., rheumatoid arthritis [2] and stroke [3]) may contribute to a reduction in hand dexterity, grip strength, and the general range of motion needed for completing daily activities [4,5,6].

In a clinical setting, several standard tests are available to evaluate the decline or recovery of functions of the hand and upper extremity, such as the Fugl-Meyer assessment (FMA) [7], Wolf motor function test (WMFT) [8], and box and block test (BBT) [9,10,11]. Some of the tests, such as the BBT and Purdue Pegboard Test, have been specifically devised to explore gross- and fine-motor hand functions required in object manipulation. For instance, the BBT focuses on hand dexterity with simple and repetitive tasks consisting of grasping, transporting, and releasing small blocks [9]. A modified version, the targeted BBT (tBBT), is characterised by more closely modelled common real-world object manipulation for controlling precision aiming [12]. To date, recent advancements in virtual reality and sensing technologies have contributed to improving the sensitivity, automation, and accessibility of the BBT. Examples include digital BBT (dBBT) with depth camera detections [13] and a haptic-combined virtual reality BBT [14].

The aforementioned studies provide a useful measure of the overall performance of hand movement in the sense that they mainly utilise temporal measures during task performance (e.g., the time required to transport the blocks in tBBT). However, limited investigations have been reported on the kinematic parameters of hand movements.

**Figure 1:**
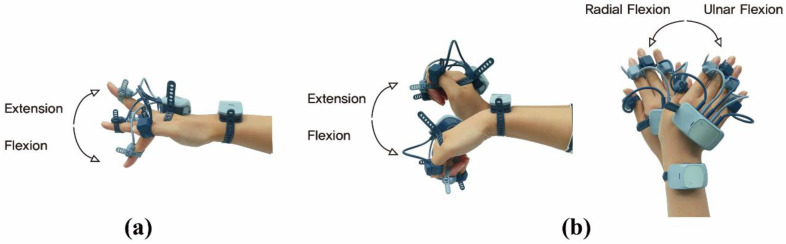
Definition of range of motion of the hand and wrist: (**a**) finger extension and flexion and (**b**) wrist flexion/extension and wrist radial/ulnar flexion.

The human hand is remarkably complex, comprising 27 bones that are controlled by 39 muscles [15]. During the course of the hand movement, an active range of motion (RoM) of the joints in the fingers, wrist, and forearm are generated. Figure 1 shows the RoMs of the hand flexion/extension, wrist flexion/extension, and radial/ulnar flexion. In particular, extension and flexion of the thumb and index finger joints are important to grasp objects in daily activities [16].

The kinematic analysis of hand movement relies on two main approaches: optical-based [17,18] and sensor-based [19,20,21,22] systems. Optical systems (e.g., Vicon or Optotrak) offer a high tracking accuracy and are often regarded as the gold standard in motion analysis [22]. However, the systems can be restricted to assess fine-motor joint movements, such as the fingers, because they are highly sensitive to alterations in the set-up, relating to marker placements, illuminations, and spatial conditions, which require considerable costs and expertise [20].

Recently, the advent of sensors and wireless communications have expedited kinematic measurements of hand movements. Connolly et al. [19] developed a wireless smart glove system to assess the deficiency of hand movements in patients with rheumatoid arthritis. The system uses multiple inertial measurement units (IMUs) placed on a stretchable, wearable glove and measures the joint angles and velocities of the finger and thumb movements in dynamic hand use conditions. Salchow-Hömmen et al. [20] investigated the measures of finger segment orientation and fingertip positions to better track and quantify the motion of the hand and its interaction with objects. With ergonomic considerations and new estimation algorithms of the system, an IMU-based modular hand sensor system was devised. However, they evaluated the system with simple object manipulation tasks, such as pinching and grasping wooden blocks, which are not realistic scenarios or standardised tasks. Lin et al. [21] further introduced a sensorised glove with IMUs and force-sensing resistors. Measuring hand kinematics and fingertip force together can yield additional information. In particular, when dealing with tasks related to grasping or lifting an object, the force sensors that must be worn on the fingertips are bulky and lead to inaccuracy with the motor-impaired hand.

Despite the growing popularity of IMU-based wearable systems for hand function evaluation, several limitations have hindered their applicability in clinical settings. First, previous studies lack empirical validity, relying on basic tasks with no connections to standard hand performance tests and a small number of subjects (e.g., two and four subjects participated in the empirical study of [21] and [20], respectively). Second, although several efforts have been made to reduce the dimensions and weights of IMU-based wearable systems [20], undesirable contacts and tangled wires observed in numerous glove- or band-type systems are still problematic. The system is considered cumbersome and limits the ability to naturally perform hand-object interactions. Furthermore, considering that chronic diseases—stroke and arthritis—are likely to be accompanied by hand joint inflammation and deformity, current non-customizable wearable systems report critical limitations for use in the clinical environment.

In this regard, the aim of this study was three-fold. The first aim was to propose a novel clip-on IMU system that is applied to the fingers and wrists. We considered ergonomic aspects to fit diverse hand dimensions and deformations. In addition, a light modular design can sense target finger movement selectively with minimum contact on the bare hand to avoid possible disruptions in hand-object manipulation. The second aim was to determine the performance and kinematic parameters derived from the proposed system to assess fine-motor hand functions. Although previous sensor-based approaches attempted to identify specific kinematic parameters of hand movement, including assessment of angular velocity and RoMs, current clinical hand assessments still resort to temporal measures during the performance of a task (e.g., average time to execute). Thus, this study aimed to investigate the integrative approach of assessing hand functions in clinically relevant conditions, which relates to the third aim of the study. Finally, we aimed to better characterise age-related differences with regard to the performance and kinematics of hand functions using a standard hand dexterity test, the tBBT. The tBBT is a performance outcome measure of hand dexterity introduced by Kontson et al. [12], which models real-world object manipulation scenarios well. 

## 2. Materials

### 2.1. Hardware Design: Clip-On IMUs System

**Figure 2:**
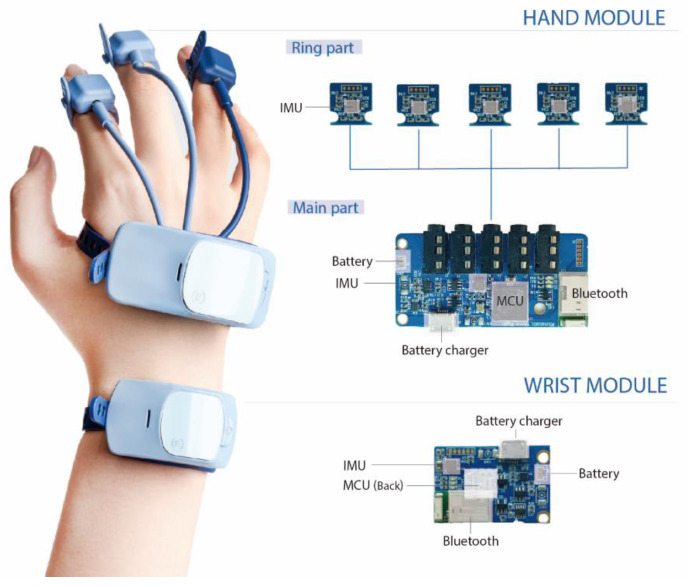
System architecture: the hand and wrist modules.

A primary challenge of the hardware design was to precisely measure hand movements by developing lightweight, mini-sized IMU modules. Figure 2 shows the system architecture of the proposed clip-on IMU system, consisting of hand and wrist modules. The hand module comprises a main part and a set of five ring parts. The main part is comprised of a main controller unit (MCU) (STM32F407IEH6, STMicroelectronics, Geneva, Switzerland), a nine-axis IMU sensor (a three-axis accelerometer, a three-axis gyroscope, and a three-axis magnetometer) (MPU9250, InvenSense, San Jose, CA, USA), and a Bluetooth v2.0 + EDR module (PAN1321i, Panasonic, Osaka, Japan). The system operates on a battery (3.7 V, 250 mAh), which can be recharged with an embedded charger chip (BQ24040, Texas Instruments). The main and ring parts are wired to provide power and transmit data. The user can simply plug in and out (up to five ring parts in each hand) so that the desired finger joints can be selectively analysed. The wrist module is identical to the main part of the hand module. A wireless connection between the wrist module and hand module is supported through Bluetooth.

**Figure 3:**
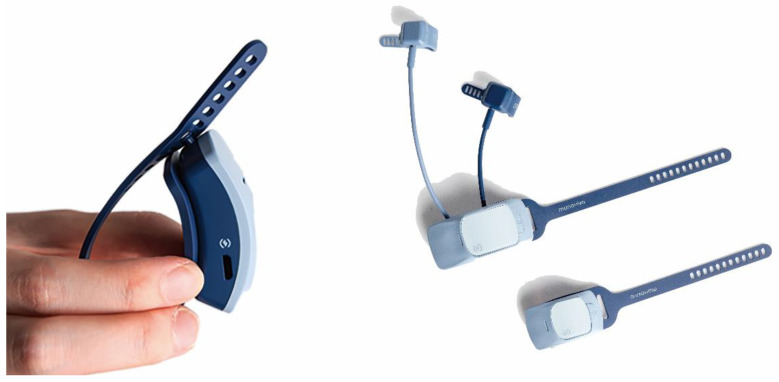
Design features: customization, flexibility, and extensibility.

The total weight of the hand module was 68 g, including 38 g for the main part. Each ring part with a fingertip sized IMU weighed 6 g. The weight of the wrist module was 29 g. These small, relatively light-weight modular designs allow compact dimensions with minimum encumbrance of the hand workspace and inter-finger interference to reduce undesirable contact on the bare hands and increase the precision of hand-object interactions. As shown in Figure 3, the system can be universally clipped onto the fingers and wrist regardless of hand dimensions and deformities. All modules are versatile enough to be placed on either the left or right hand. Moreover, soft and elastic materials provide additional comfort and flexibility in hand movements. In terms of extensibility, a clip with a flexible band-type design and fingertip-sized IMU modules with separation allowed the placement of up to five finger modules for each hand. Other biomedical sensors can be extended in connection with the main part of the hand module for further analysis.

### 2.2. Joint Angle Estimation Algorithm

To estimate the joint angle based on inertial sensors mounted with respect to the fingers and wrist, we first calculated the Euler angles of each IMU sensor. There are issues concerning the validity and reliability of the measurements obtained from the IMUs [23,24]. With the aim of reducing possible integration errors using magnetometer data (geomagnetic field) and accelerometer data (gravity acceleration), this study employed the gradient-descent algorithm proposed by Madgwick [24]. Empirical verification of the Euler angles, roll, pitch, and yaw of the IMU system was then conducted under static and dynamic conditions. 

For the static condition, the Euler angle was measured three times at 30°/45°/60° inclinations and compared with reference to a digital inclinometer (BevelBox, TekcoPlus Ltd., Kowloon, Hong Kong). Table 1 listed the Euler angles for each angle. The mean errors are 0.41° (phi), 1.13° (theta), and 0.68° (psi).

The Euler angle in the dynamic conditions was verified in our previous studies [25,26].

Compared to the reference angle obtained in a reflective marker-based 3D infrared system Vicon, the root mean square error (RMSE) was 2.56° at roll, 1.72° at pitch, and 1.08° at yaw. In summary, our clip-on IMU system was able to measure finger and wrist joint angles with an average RMSE error of 1.78° under dynamic conditions.

**Figure 4:**
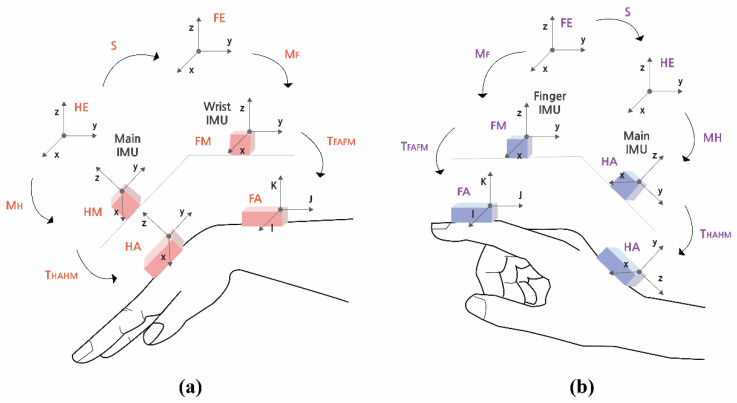
Algorithm for joint angle estimation between adjacent segments: (**a**) the wrist and (**b**) finger.

Figure 4 shows anatomical joint angle estimations with a pair of IMUs. We used the definition and equations from the study of Brennan et al. [27], which resolved the joint angle estimation error owing to the physically mounted IMU sensors onto body segments.

Figure 4a shows the forearm anatomical (FA) and hand anatomical (HA), and the sensors attached to the anatomical positions are represented by HM (hand measurement) and FM (forearm measurement). $T_{FAFM}$ and $T_{HAHM}$ are time-invariant rotation matrices between the actual position of the IMUs and the anatomical position (Equation (2)). The parameter S aligns the forearm and hand positions in accordance with the angle θ. The time-dependent orientation matrices $M_{H}$ and $M_{F}$ describe the orientation matrix of the forearm and hand with respect to the earth frames FE and HE, respectively. $T_{HAFA}$ which is ultimate matrix representing the joint angle between the main IMU on the hand and the IMU on the wrist is expressed in Equation (1). The relationship between anatomical angles and cardan rotation matrix is the same as in Equation (3) (α: Flexion/Extension, β: Ulnar/Radial flexion, γ: rotation).

(1) $$T_{HAFA}=T_{HAHM}M_{H}SM_{F}^{-1}T_{FAFM}^{-1}$$

(2) $$T_{HAHM}=T_{FAFM}=\left[\begin{array}{ccc} -1 & 0 & 0\\ 0 & 0 & 1\\ 0 & 1 & 0 \end{array}\right]$$

(3) $$T_{HAFA}=\left[\begin{array}{ccc} \cos\beta \;\cos\gamma & -\sin\gamma \;\cos\beta & \sin\beta \\ \cos\gamma \;\sin\beta \;\sin\alpha \;+\;\sin\gamma \;\cos\alpha & \cos\alpha \;\cos\gamma \;-\;\sin\alpha \;\sin\beta \;\sin\gamma & -\;\cos\beta \;\sin\alpha \\ \sin\gamma \;\sin\alpha \;-\;\cos\gamma \;\sin\beta \;\cos\alpha & \sin\gamma \;\sin\beta \;\cos\alpha +\cos\gamma \;\sin\alpha & \cos\beta \;\cos\alpha \end{array}\right]$$

The $T_{HAFA}$ matrix terms were used to calculate the two joint angles of flexion/extension and ulnar/radial flexion, as shown in Equations (4) and (5), respectively. The same calculation method was applied to obtain the finger flexion/extension angles, as shown in Figure 4b and Equation (4)

(4) $$Flexion/Extension\left(\alpha \right)=\tan^{-1}(\frac{-T_{HAFA}\left(2,3\right)}{T_{HAFA}\left(3,3\right)})$$

(5) $$Ulnar/Radial\;flexion\left(\beta \right)=\sin^{-1}\left(T_{HAFA}\left(1.3\right)\right)$$

## 3. Experimental Protocol

The empirical study is designed to increase the ecological validity of the clip-on IMU system and to examine the potential to characterise age-related differences in hand functions. We extracted a set of performance and kinematic parameters using the clip-on IMU system to provide a more comprehensive understanding of fine-motor finger and wrist movements.

### 3.1. Data Collection

Thirty-four healthy adults (18 women, 16 men) aged 20–89 years participated in the study. Participants were then divided into two groups, younger and older adult groups. The younger group included 18 healthy adults (age 26.2 ± 3.0, range: 20–31 years). The older group included 16 healthy adults (age 82.63 ± 3.4, range: 75–89 years). All subjects were right-handed. Participants were briefed about the purpose of the study and signed informed consent before the procedure. The study was reviewed and approved by the Hanyang University Institutional Review Board (HYI-18-142-2).

### 3.2. Task and Procedure

All subjects were asked to perform the tBBT. The tBBT [12] involves precise and rapid movement of the fingers and wrist, while grasping, transporting, and releasing a wooden block. In comparison to the standard BBT [11], which requires transport of as many blocks as possible in 1 min, the tBBT requires transport of a total of 16 blocks as quickly and accurately as possible using an ordered selection and a controlled placement of the block [12]. 

**Figure 5:**
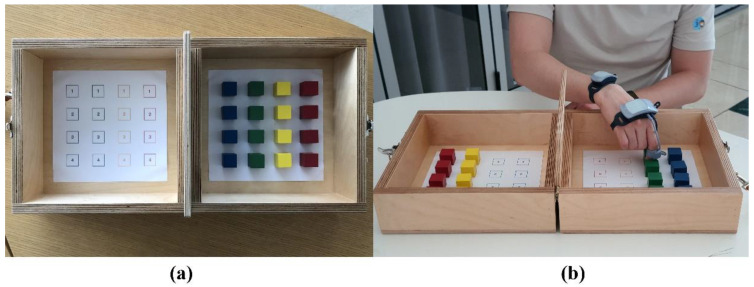
Experimental set-up: (**a**) configuration of the targeted box and block test and (**b**) positioning of the clip-on IMU-system to assess hand and wrist movements.

Figure 5a shows the configuration of the tBBT. It consists of 16 blocks, a two-sided box, and one partition in the middle of the box. In the test, subjects are asked to be seated in a chair and then transport each block over the partition, starting with the innermost left block and placing it on the right box in the same position as the left box. The dominant hand should cross the partition in each transport. During the tBBT, all the subjects were required to clip our IMU system onto their fingers and wrist. Each ring part was placed on the proximal phalanx of the thumb and the middle phalanx of the index finger to minimise interference while grasping a block, as shown in Figure 5b. The subjects were asked to complete the test three times. 

### 3.3. Parameter Extraction

Two performance parameters and four kinematic parameters were extracted. One of the performance parameters is the total time required to transport 16 blocks to an assigned grid. The other is the coefficient of variation (CV) of the time to transport 16 blocks. This can be used to assess whether each transport movement is applied regularly in the test. There are two kinematic parameters for the finger movement (flexion/extension RoMs of the thumb and index finger) and two for the wrist movement (flexion/extension RoMs and ulnar/radial flexion RoMs). The terms of hand movement used is shown in Figure 1.

### 3.4. Statistical Analysis

Six parameters were used for the statistical analysis. Values greater or less than the range of 1.5 quartiles in the upper and lower quartiles based on the median in the total data were confirmed using boxplots and excluded from the analysis [28]. Group differences were assessed with independent t-tests if the data satisfied a normal distribution according to the Kolmogorov–Smirnov test. If not, we employed the Mann–Whitney test. Differences were considered significant at * p < 0.05. All statistical analyses were conducted using IBM SPSS Statistics 24.0 for Windows (IBM, Armonk, NY, USA).

## 4. Results

### 4.1. Performance Parameter Analysis

**Figure 6:**
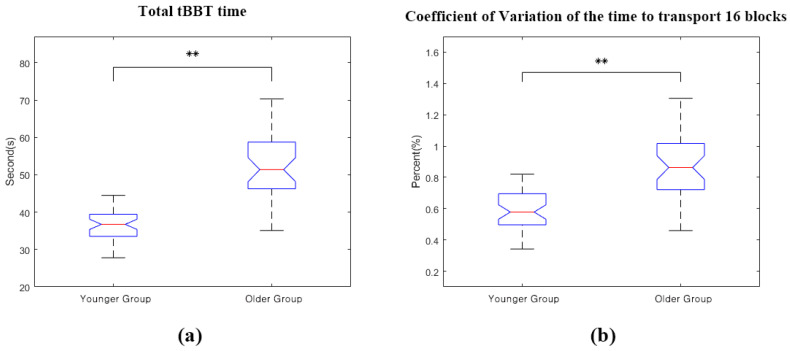
Performance parameters in the tBBT by age: (**a**) the total tBBT time and (**b**) Coefficient of Variation of the time to transport 16 blocks (** *p* < 0.01).

The performance parameters in the tBBT were compared by age (Figure 6). The performance parameters in the tBBT were compared by age (Figure 6). The age difference showed a significant difference in the time to transport the 16 blocks (t-test, ** *p* < 0.01). The older group required more time to complete the tBBT (Mean = 51.9 s, SD = 10.6 s) than that in the younger group (Mean = 36.6 s, SD = 5.1 s). The CV of the time to transport a block during the tBBT showed higher variability in the older group (Median = 0.86%, IQR = 0.30%) than that in the younger group (Median = 0.58%, IQR = 0.20%), with significant values of ** *p* < 0.01 (Mann–Whitney test).

### 4.2. Kinematic Parameters Analysis

**Figure 7:**
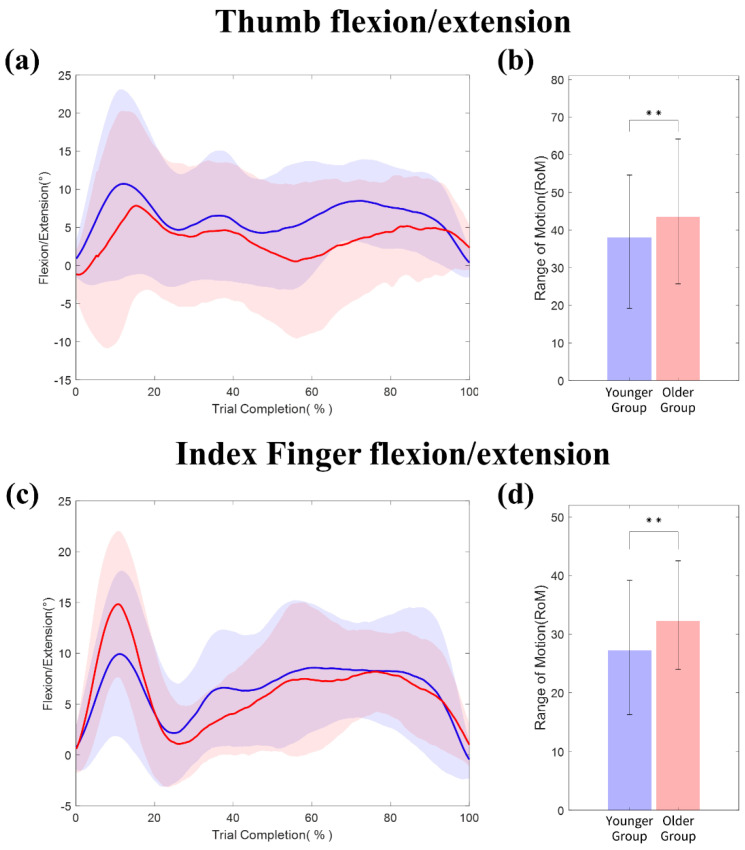
Kinematic trajectories and the RoM of the thumb and index finger for the younger (blue) and older adults (red): (**a**) the thumb and (**c**) index finger of the flexion/extension trajectories by age and (**b**) the thumb and (**d**) index finger flexion/extension RoM by age.

**Figure 8:**
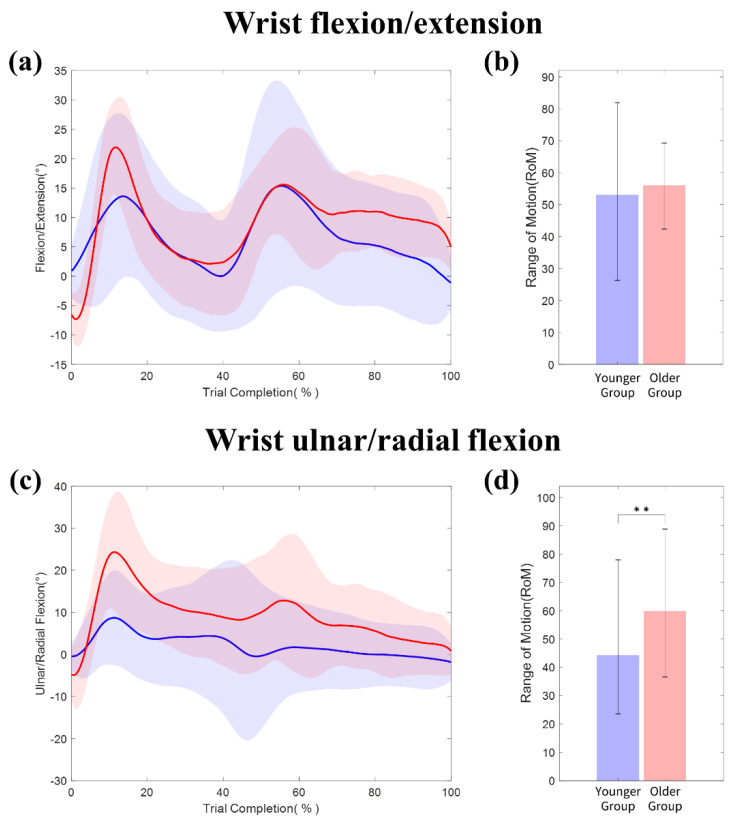
Kinematic trajectories and RoM of the wrist for the young (blue) and older adults (red): (**a**) wrist flexion/extension trajectories by age (**b**) wrist flexion/extension by age and (**c**) wrist ulnar/radial flexion trajectories by age; (**d**) wrist ulnar/radial flexion RoM by age.

Figure 7 and Figure 8 compare the kinematic trajectories and RoM of the fingers and wrist by age. The kinematic trajectories are shown as a function of percent trial completion. The starting point of all kinematic data was set to zero as the initial hand posture of each subject was variant. The percent trial completion represents a single sequence of the tBBT, consisting of grasping, transporting, and releasing a bock, and moving back to the initial position, which are proportionally distributed.

**Finger movements**: Figure 7a,c show the kinematic trajectories for flexion/extension of the thumb and index finger. In initial object grasping, the thumb and index finger flexion markedly increased compared to the remaining trial completion. In addition, the change in the thumb is slightly greater than that in the index finger. Significant differences (t-test, ** *p <* 0.01) in the RoM of the thumb by age were observed for the thumb flexion/extension: young adults (Mean = 36.2°, SD = 8.8°) vs. older adults (Mean = 44.2°, SD = 9.9°). Similarly, the older adults significantly increased the RoM of the index finger (Median = 32.2°, IQR = 8.5°) in comparison to the younger adults (Median = 27.2°, IQR = 7.7°) (Mann–Whitney test, ** *p* < 0.01); see Figure 7b,d. The results reveal a larger use of joint angles in the older group than that in the younger group to complete the same task.

**Wrist movements**: The kinematic trajectories of the wrist flexion/extension and radial/ulnar flexion are shown in Figure 8a,c, respectively. Compared to the kinematic trajectories of the fingers, we observed two peaks for the wrist movement. The first one is the same as the initial movement to grasp a block (i.e., 15–20% trial completion). The following occurred immediately before the release of the block (i.e., 55–60% trial completion). The peak flexion of the wrist occurred slightly earlier than that of the hand.

Both groups had a high RoM for the wrist flexion/extension in Figure 8b: young adults (Median = 53.0°, IQR = 28.9°) vs. older adults (Median = 56.0°, IQR = 12.1°). No age difference was reported in this case (Mann–Whitney test, *p* = 0.248). In Figure 8d the RoM of the wrist radial/ulnar flexion in the younger group (Median = 44.2°, IQR = 21.0°) was significantly lower than that in the older group (Median = 59.2°, IQR = 22.2°). The differences were statistically significant at ** *p* < 0.01 (Mann–Whitney test).

## 5. Discussion

### 5.1. Novel Clip-On IMU System for Fine-Motor Hand Function Assessment

This study proposed a novel clip-on IMU system that can be applied to the fingers and wrist and provides a comprehensive understanding of fine-motor hand function. The development of the clip-on IMU system presented in the study focussed on three design features: customisation, flexibility, and extensibility. The customisable clip-type design can be fitted regardless of hand dimensions, inflammation, and deformity of the joints. Each clip is manufactured using a stretchable, flexible material that yields extra comfort and minimum disruptions to perform hand-object manipulation. There are two substantial benefits for clinical use settings. One is to target patients with a broad spectrum of conditions affecting the hand and wrist, such as rheumatoid arthritis, other inflammatory arthropathies, and age-related dexterity declines. The other is to reduce the impact of the system on hand movements, while preserving the sense of touch.

Similarly, earlier studies have addressed issues related to the flexibility and practicability of the sensorised glove systems for use in the clinical environment. The glove proposed by Connolly et al. [19] was designed to help quantify the RoMs of each finger joint and monitor patient progression during the arthritis rehabilitation process. Despite their attempts to construct stretchable printed circuit boards for a flexible glove, the glove type was bulky as it covered the entire hand and was incapable of the sense of touch. An experimental validation was not conducted in the context of hand-object manipulation. Lin et al. [21] further combined the IMUs with force-sensing sensors to accurately measure hand–object interactions. Providing kinematic data and fingertip force yields additional information when patients conduct hand-object manipulation tasks. These advanced features have substantial benefits for assisting in the clinical environment. There are challenges with regard to the trade-off between a compact design and functionality. Unlike gloves, Salchow-Hömmen et al. [20] attempted to preserve a full sense of touch by placing mini-IMU sensors on the back of the hand and fingers, which are fixed with skin-friendly tape. This design feature resulted in a weight reduction, compared to the aforementioned glove system weighing 70 g, according to [21], as well as our proposed system. To the best of our knowledge, taping sensor strips to fingers might function well in laboratory settings; however, this taped system could fall apart after multiple trials. The practicability and reusability of the taped system in clinical settings remain unsolved.

One of the important advantages of our system is its modularity and extensibility. The finger parts (6 g each) and wrist parts (29 g) can be removed or attached to the main part (38 g) according to the joints of interest. The considered systems [19,20,21] could be compared underlying the usability and ecological validity of the implementation in clinical environments.

In the following discussion, we first address the value of the integrative parameters of the performance and kinematics of hand functions and the joint use of the standard hand dexterity test, tBBT. Subsequently, the empirical findings obtained for characterising the age-related differences in hand functions are examined as they indicate the potential benefits of clinical trials.

### 5.2. Integrative Parameters of the Performance and Kinematics of Hand Functions

Several previous contributions for IMU-based hand motion tracking systems have significantly enhanced the accuracy of the system [19,20,21]. However, only a small number of studies have conducted evaluations with human subjects. For instance, the Salchow-Hömmen’s [20] system was designed and tested with four able-bodied adults using functional hand motions, such as grasping or pinching small objects. However, this was not a clinically relevant condition. Few exceptions were found in the studies of Connolly et al. [19] and Lin et al. [21]. Their empirical studies were validated among patients with rheumatoid arthritis or mild hand movement disorder. However, both studies had a limited sample size (nine subjects [19] and two subjects [21]), which returned results with low ecological validity.

These shortcomings are well reflected in this study. We first improve the hand function assessment in the tBBT, which can provide a clinically valid and reliable assessment with the parameters of performance and kinematics of grasping, transporting, and releasing blocks. Compared to the aforementioned studies, a larger sample size (18 young and 16 older healthy adults) was considered. In addition to the total tBBT time, our system provided a more specific assessment of hand functions. For instance, the CV of the time in the tBBT determined movement variability in time. Four kinematic parameters, including the kinematic trajectories and RoM for the fingers and wrist, were developed to reflect motion smoothness and efficiency during task performance.

Similarly, few digitalised BBTs [13,14] have attempted to provide additional kinematic information in the BBT, using a depth camera [13] or a 3D virtual environment that allows spatial visualisation and operation [14]. However, considering some known disadvantages of the optical-based approach in Section 2, earlier investigations are unsuitable for measuring fine-motor hand movement in the BBT, which involves precise and rapid movement of the fingers.

In addition, the joint use of the finger and wrist IMU sensors shows interesting potential for the advent of a fine-motor hand movement analysis. Nonetheless, some believe that wrist-worn sensors only relate to gross-motor arm movements and provide inaccurate information for fine-motor hand movements [29]. In our study, the wrist module helps segment kinematic trajectories by capturing the movements of the hand and fingers relative to the wrist (see Figure 7 and Figure 8). This information can be used for a better understanding of the dynamic movements of the finger and wrist joints.

### 5.3. Age-Related Differences in Hand Functions

The main finding regarding hand movement performance in the tBBT was that the older group was significantly slower and showed higher movement variability than the younger group, as indicated by longer total tBBT time and greater CV of the time in the tBBT. The results on the total performance time are consistent with previous reports of age-related declines in standard BBT [10,13]. The CV of the time in the tBBT suggests additional information regarding hand movement on whether subjects chose to optimise for accuracy (e.g., minimising block placement errors) or speed [30]. This parameter can be particularly useful in common daily activities, such as picking-up and releasing mugs, requiring precision aiming and object manipulation.

The age-related varied kinematic trajectories and the RoM by the type of finger and wrist movements are evaluated. Overall, the older group required a significantly larger RoM at the thumb, index finger, and wrist when compared to the younger group (** *p* < 0.001), with one exception. The flexion/extension RoM of the wrist was indifferent to the groups. We observed that some of the subjects were likely to rely on more proximal joints, such as the shoulder and elbow. This could be partially attributed to individual habits in transferring movements. A similar pattern was observed in the study of virtual reality-based BBT [14]. However, this should be interpreted with caution, as our study did not collect relevant kinematic parameters at the elbow and shoulder.

Larger RoMs imply less efficient movement strategies for individual tasks. Our study provides evidence of age-related deficits in the efficiency of the tBBT. This finding is presented for the first time in a unique clinical context using a novel IMU-based system.

Another interesting finding was obtained in the kinematic trajectories of the fingers and wrist. During a single action of the tBBT, the finger extension peaked when the block was picked up and remained constant until the subject placed the box on the opposite side of the partition and moved back to the initial position. In comparison, two peak angles were observed at the wrist joint. The subjects started to flex the wrist as they approached a block, and the wrist gradually extended during the block movement, before flexing again as the block was released. The latter finding is consistent with the results of a previous study by Kontson et al. [12], which examined the kinematics in upper body movements during the tBBT. However, as the data in study [12] were collected from an optical system with markers (e.g., Vicon), there were limited investigations of fine-motor hand movement, such as kinematics at the fingers and wrist, which is relevant for the tBBT. Unlike the previous approach, our novel IMU-based system allowed us to obtain detailed information related to the kinematic chain between the finger and wrist. One of the findings was that the peak angle of the wrist was first, followed by those of the thumb and index fingers.

## 6. Conclusions

This study proposed a novel clip-on IMU system applied to the fingers and wrist to assess fine-motor hand movements in clinical settings. Empirical studies were conducted to verify the system accuracy in static and dynamic use conditions, and second, to examine its potential to characterise age-related decline in hand functions.

Several performance and kinematic parameters were extracted from our IMU system to comprehensively understand the hand movements, which are of paramount importance in performing the instrumental activities involved in daily living. Although the standard hand manual dexterity test (i.e., the tBBT) provides a useful measure of overall performance, it has not provided specific components of hand movement that impact speed, variability, and efficiency with advanced age.

Our findings provide some insight by demonstrating that the older group tended to show decreased time completion and higher variability during task performance and additional kinematic parameters, such as a larger RoM at the fingers and wrist, compared to the younger group. The kinematic trajectory analysis showed how the RoM of the fingers and wrist changes with specific hand functions, such as grasping, transporting, and releasing a block.

There is relative preservation of gross movements and a decline in fine manipulation in heathy aging [30]. Our clip-on IMU system and empirical validations successfully highlight a fine-motor control perspective that might be useful in future rehabilitation practice.

Future studies are needed to increase the ecological validity of the hand dexterity tests in a larger sample and with investigations of hand-object manipulation relevant to real-life activities. In addition to the simple performance-based standard test, our system can provide clinically validated advanced performance and kinematic parameters that contribute to the decline in manipulation. We can further extend and advance the current understanding by establishing normative ranges in different age groups to help guide clinicians to differentiate between decline in hand movement attributable to normal ageing and that attributed to pathological states. Future studies should address more relevant daily living activities that include the hands and real-world objects in unimanual and bimanual tasks. Some bimanual activities of daily living require speed and precision (e.g., typing computer keyboards) or forces (e.g., opening containers). Although the current IMU system can assess the bimanual tasks with both hands, some challenges remain. In addition to the ergonomic aspects of the wearable system (e.g., dimensions, weights, undesirable contacts), the system is further combined with force sensors, allowing for accurate hand function assessment, particularly in object manipulation.

## Figures and Tables

**Table 1:** Comparison of the Euler angles for each axis in the static condition.

Reference	Axis	1st	2nd	3rd
30°	Roll	29.8°	29.5°	29.5°
	Pitch	29.2°	29.2°	29.2°
	Yaw	30.0°	30.5°	30.1°
45°	Roll	44.8°	44.4°	44.4°
	Pitch	43.7°	43.9°	43.9°
	Yaw	43.8°	44.5°	44.6°
60°	Roll	59.6°	59.2°	59.3°
	Pitch	58.6°	58.5°	58.6°
	Yaw	59.6°	59.2°	58.5°
